# Enhancing the SIB Literature Services (SIBiLS) with annotations to support biocuration

**DOI:** 10.1093/database/baag034

**Published:** 2026-09-22

**Authors:** Déborah Caucheteur, Alexandre Flament, Julien Gobeill, Emilie Pasche, Pierre-André Michel, Luc Mottin, Jeevanthi Liyanapathirana, Anaïs Mottaz, Patrick Ruch

**Affiliations:** HES-SO Genève, Tambourine 17, 1227 Carouge, Switzerland; SIB Swiss Institute of Bioinformatics, Tambourine 17, 1227 Carouge, Switzerland; HES-SO Genève, Tambourine 17, 1227 Carouge, Switzerland; SIB Swiss Institute of Bioinformatics, Tambourine 17, 1227 Carouge, Switzerland; HES-SO Genève, Tambourine 17, 1227 Carouge, Switzerland; SIB Swiss Institute of Bioinformatics, Tambourine 17, 1227 Carouge, Switzerland; HES-SO Genève, Tambourine 17, 1227 Carouge, Switzerland; SIB Swiss Institute of Bioinformatics, Tambourine 17, 1227 Carouge, Switzerland; HES-SO Genève, Tambourine 17, 1227 Carouge, Switzerland; SIB Swiss Institute of Bioinformatics, Tambourine 17, 1227 Carouge, Switzerland; HES-SO Genève, Tambourine 17, 1227 Carouge, Switzerland; SIB Swiss Institute of Bioinformatics, Tambourine 17, 1227 Carouge, Switzerland; HES-SO Genève, Tambourine 17, 1227 Carouge, Switzerland; SIB Swiss Institute of Bioinformatics, Tambourine 17, 1227 Carouge, Switzerland; HES-SO Genève, Tambourine 17, 1227 Carouge, Switzerland; SIB Swiss Institute of Bioinformatics, Tambourine 17, 1227 Carouge, Switzerland; HES-SO Genève, Tambourine 17, 1227 Carouge, Switzerland; SIB Swiss Institute of Bioinformatics, Tambourine 17, 1227 Carouge, Switzerland

## Abstract

Text-mining techniques are essential tools to efficiently access information within an increasing number of publications. The SIB Literature Services (SIBiLS) are dedicated to the text-mining and biocuration communities. SIBiLS features an extensive repository comprising ~40 million abstracts from MEDLINE, 8 million full-text articles accessible through the National Library of Medicine (NLM) via PMC, 29 million supplementary data files, and ~1 million taxonomic treatments sourced from Plazi and other contributors. The full-text articles are also containing original Open Access journals, which are not commonly indexed by PMC but relevant for research and scientific communities, the so-called PMC + collection. SIBiLS is thus a superset of the NLM collections. Several text-processing strategies have been implemented (e.g. tokenization, hyphenation, concept normalization), leading to the enrichment of SIBiLS with automatic annotations based on term mapping from ~30 ontologies. The aim is to improve literature triage and curation time by automating the extraction and organization of relevant information from large volumes of scientific texts. The annotation process has generated over 16 billion annotations. One of the goals of this enhancement is to improve the recall of rare contents, including genomic variants and information on rare diseases. It is also used in various contexts, including studying biotic interactions and curating genomic variants through dedicated front-end applications such as the BiotXplorer and Variomes. Annotations are performed using the BioC standard. By combining JATS and BioC, SIBiLS enables curators to perform high-precision evidence tracking at any textual representation levels via an original annotation schema (e.g. IDs, onto-terminological sources, provenance). An assessment of the annotation quality has been carried out for the different annotated entities. The average annotation accuracy is approaching 90% but exhibits high variation depending on both the entity and the source vocabulary.

## Introduction

### Overview of SIBiLS

SIBiLS (Swiss Institute of Bioinformatics Literature Services) is a resource designed to offer effective information retrieval and facilitate analysis of scientific literature for researchers in life sciences [1]. It integrates state-of-the-art text mining and natural language processing (NLP) techniques to navigate the ever-growing volume of biomedical and life sciences literature. By providing tools for automated literature annotations and knowledge extraction, this resource accelerates the process of uncovering insights and identifying relevant scientific information. While various NLP methods have been developed to perform named entity recognition (NER) and normalization [2–4], the SIBiLS annotation pipeline reported here uses simple pattern matching strategies for sake of scalability. Indeed, the full annotation of our collections (∼nearly 80 million documents) can be performed in a few hours on a standard server.

### SIBiLS collections

The essence of SIBiLS is the aggregation of document collections from different sources, each providing a unique perspective and type of information that contributes to a more complete understanding of the biomedical and biodiversity literature. MEDLINE citations provide bibliographic information, full texts (from PMC or third-party publishers) provide in-depth content, supplementary data provide additional experimental details [5], and Plazi treatments provide taxonomic descriptions. By aggregating these collections and annotating documents with reference vocabularies daily, SIBiLS enables a more comprehensive and detailed exploration of the scientific literature. It is poised to streamline literature triage and seamlessly integrate into any curation workflow, leveraging state-of-the-art analytical (e.g. question answering) and scalable technologies such as MongoDB and Lucene ElasticSearch, which adapt to the demands of big data, and making data available in BioC format via FTP.

### Annotations

Generated automatically on a daily basis, the annotation process aims to enhance search effectiveness by enriching content with meaningful metadata. It processes various collection elements, including text, tables, OCR-processed images, and supplementary data files [6], using 30 vocabularies or ontologies to ensure comprehensive and accurate indexing. Available in the BioC format like the original data, annotations are structured in several fields like described in the “Material and methods” section.

### Services and API

SIBiLS provides access to these contents and annotations via RESTful APIs, enabling users to fetch annotated content (fetch API), search within annotated collections (search API), and also ask questions to retrieve papers of interest (question answering API). Detailed documentation on endpoints, parameters, and data formats is available on the services homepage (https://sibils.org/), along with examples to demonstrate how to interact with the services.

In addition, multiple services under the umbrella and in relation with SIBiLS are available to users depending on their needs. Each of these services exploits the content and annotations processed into SIBiLS. Variomes (https://variomes.sibils.org/) [7, 8] is an application specifically designed to support the exploration and prioritization of human genetic variants. The system offers dual functionality: it serves as a literature triage tool and enables the prioritization of variants to streamline the detection of clinically actionable variants. Another service is named BiotXplorer (https://biotxplorer.sibils.org/) [9], a tool that scans scientific literature to identify pairs of species that may share a biotic relationship. For each detected interaction, it provides one or more relevant excerpts from the literature. This enables users to uncover new biotic interactions and gain insights into how these relationships are formed.

This publication aims to assess the quality of the annotations processed within SIBiLS and leverage the findings to identify potential improvements. These enhancements are intended to optimize the system and provide better services to users.

## Material and methods

### A document processing and publication workflow

#### Backend—collect and pre-processing of documents

**Figure 1:**
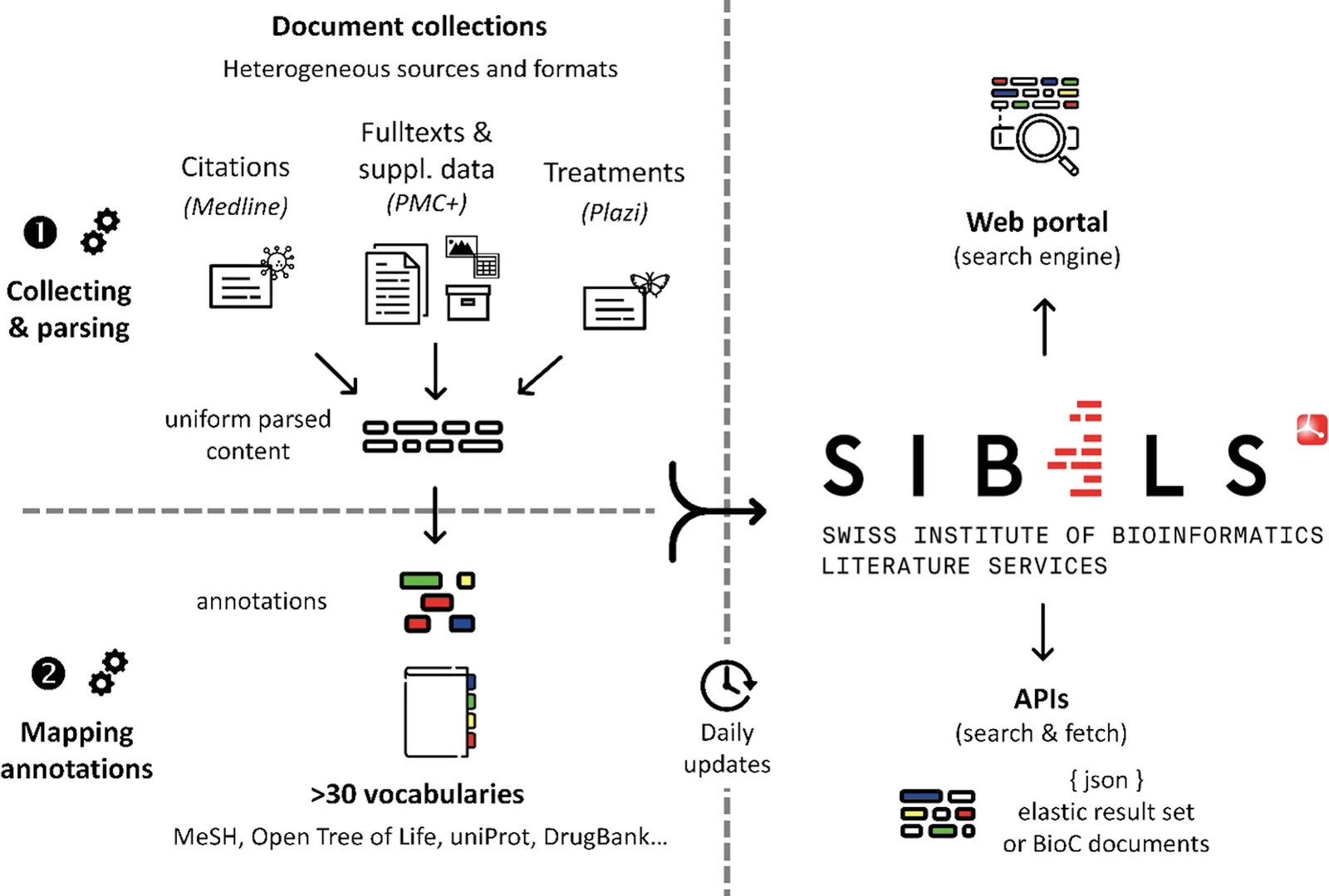
Overview of the SIBiLS Literature Services workflow. The figure illustrates the backend and frontend processes of the SIBiLS (Swiss Institute of Bioinformatics Literature Services system).

Aggregating collections means collecting data in different formats from different sources. There are two main autonomous processes in the SIBiLS backend (Fig. 1). In the process of the ‘Collecting and Parsing’ pillar, the documents are collected and updated on a daily basis from various sources: the FTP of the National Library of Medicine, which is harvested by SIBiLS, or the FTP of SIBiLS, where Plazi and third-party publishers upload their documents. Local parsers then extract the text from the various documents using specific strategies, whether these are articles in JATS XML, or supplementary data contained in very heterogeneous file formats: images (JPG, PNG files…), spreadsheets (Excel or CSV files), MS Word files, XML, HTML, or TXT. The result of this process is a unique and normalized representation of the documents, where contents are identified with high granularity at sentences or even offset levels using BioC. The BioC format is an XML-based framework for sharing and processing biomedical texts. It structures text, annotations, and relations to maintain consistency across biomedical text mining applications, promoting data exchange and collaboration. The ‘infons’ section is a key component in BioC used to store metadata. It enhances flexibility by allowing customized information to be embedded within the BioC structure, used in our case to store contents of annotations (Fig. 3). This BioC format ensures that the ‘Mapping Annotations’ process operates on a standardized data format, allowing for seamless integration of different annotation versions—whether refining matching algorithms or introducing new vocabularies—without requiring full reprocessing of collections. The extracted content is then annotated using over 30 vocabularies (see Table 1), including MeSH (Medical Subject Headings), Open Tree of Life, UniProtKB/SwissProt, DrugBank, etc., with daily updates to maintain accuracy and relevance.

**Table 1:** Terminologies and ontologies used in SIBiLS.

Biodiversity	Ecology	Agrovoc [10]
		ENVO [11,12]
		FLOPO [13]
		Plant Ontology (PO) [14]
		Relation Ontology (RO) (enriched) [15]
	*Species*	ICTV [16]
		Mamman Diversity Database (MDD) [17]
		NCBI Taxonomy [18]
		Open Tree of Life (OTT) [19]
	*Chemicals*	LOTUS (natural products) [20]
		PubChem (subset) [21]
Molecular biology		Cell Ontology [22]
		Cellosaurus [23]
		COVoc [24]
		Detection and Methods
		Evidence and Conclusion Ontology (ECO) [25]
		PPI PTM
		PSI-MI [26]
Genes and proteins		DisProt [27]
		Gene Ontology (GO) [28,29]
		neXtProt [30]
		UniProtKB/SwissProt [31]
Health		ATC [32]
		CHEBI [33]
		Drugbank [34]
		ICD.O.3 [35]
		MeSH [36]
		NCI Thesaurus [37]
FAIR		Accession Numbers [38]
		Affiliations (GRID) [39]
		Crossref Funder Registry [40]
		Licenses (SPDX) [41]

A total of 32 vocabularies, regularly updated, are used to create the annotations of SIBiLS, in order to improve search effectiveness and meet the needs of services like BiotXplorer or Variomes.

#### Frontend and API

**Figure 2:**
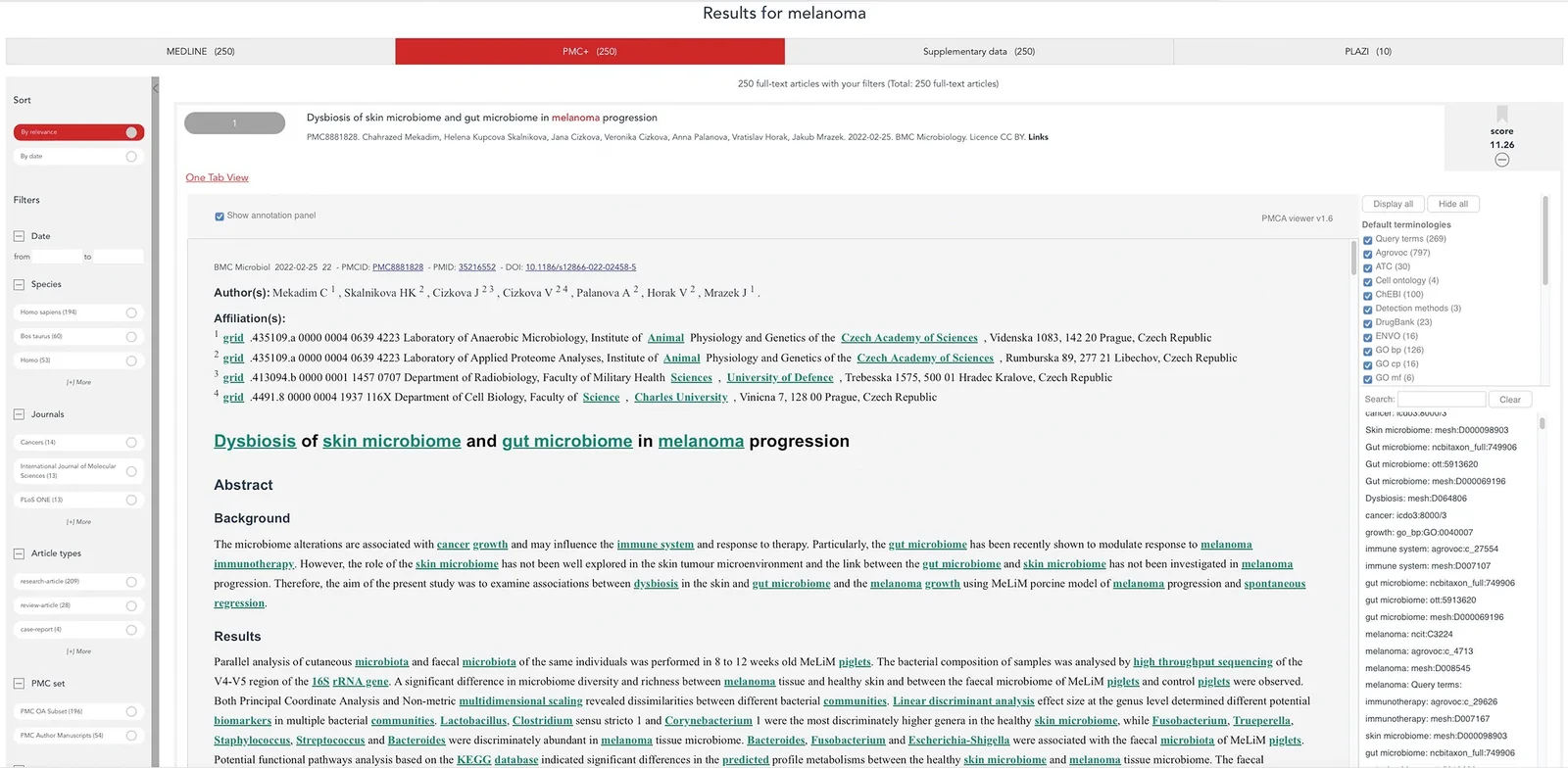
Graphical User Interface of BiodiversityPMC. This screenshot displays the graphical user interface of BiodiversityPMC, allowing access to documents enriched with annotations visually integrated into the text.

For the frontend applications, the original document representation in JATS and the automatic annotations in BioC are combined in an original viewer (https://github.com/sibils/sibils2bioc). These two standards are the pillars of BiodiversityPMC, the main search user interface for exploring the annotated literature (Fig. 2), as well as the complementary front ends: Variomes for rare variants and BiotXplorer for biotic interactions. Further, a set of RESTful APIs and an FTP server for bulk downloads are available. An RDF endpoint (currently in beta) is also available to search the annotations with SPARQL queries (https://sibils.org/sparql/).

### Vocabularies and sources for annotations

#### Terminologies used as basis for the annotation process

Our annotation process is based on the use of terminologies from different research fields. As mentioned before, historically, the SIBiLS services were dedicated to biomedical research and therefore used terminologies such as ATC and DrugBank for drugs, GO, or MeSH. For the purposes of the SVIP-O project [7] and the Variomes service (https://variomes.sibils.org/) [8], neXtProt for genes and NCI Thesaurus for diseases were particularly of interest for curators working on the platform. However, the number of terminologies has continuously grown to keep up with the SIB curation support effort, and currently, a total of 32 vocabularies are used to map entities (Table 1). The most recently added terminologies include the CelloSaurus—a newly GCB (Global Core Biodata)-labelled database for cell lines and terminologies relevant to curated biotic interactions.

We have recently integrated new terminologies into the existing framework, each addressing specific objectives to enhance the system’s capabilities across various categories:

- **Accession numbers**: The accession numbers list constructed as terminology is based on the data available on the GitHub repository linked to registry.org (https://github.com/identifiers-org/registry). A filtering of too permissive patterns was carried out manually like for the COSMIC database with the pattern *^[A-Z0-9]+$’*. From the initial 591 patterns provided via identifiers.org, 58 are kept into the terminology format. Manual insertions of three patterns were done to add GenBank identifiers and Plazi URLs to meet internal project requirements.
- **FAIR principles**: The inclusion of specialized vocabularies, such as SPDX for identifying licensing agreements and ROR for organizational affiliations, strengthens the system’s ability to extract relevant legal and institutional information. Additionally, a dedicated algorithm has been developed to identify grant numbers, enabling more efficient creation of grant-linked annotations.
- **Biodiversity**: New terminologies like FLOPO, AgroVoc, and Plant Ontology have been incorporated to provide more precise and tailored annotations, facilitating the accurate identification of taxonomic entities, ecological phenomena, and habitat characteristics.
- **Biotic Interactions**: The ROBI (Relation Ontology Biotic Interaction) is a subset of the Relation Ontology enriched by our team, specifically designed to capture biotic interactions between species via the BiotXplorer service. Its integration marks a significant advancement, enhancing the system’s ability to analyse complex ecological relationships within textual data.

#### Selecting onto-terminological entities

The initial procedure involves retrieving terminologies or ontologies in their original formats from multiple sources as detailed, see https://sibils.org/vocabularies/ for an updated list of resources. These terminologies are selected according to the needs of partners and collaborators, as well as the requirements of the projects in which the team is involved. The formatting process involves several critical components. Each identifier in the original file retains its terms (the matched word) and respective natures (if it is a preferred_term or synonym) within the JSON structure. Further, a header is added to outline the terminology’s characteristics, including version number, date of update, and formatting parameters. Across most terminologies, an exclusion filter is applied to filter out terms, typically by removing high-frequency English words. Further, terminologies may feature custom exclusion filters, tailored to specific terms through manual curation. The concept is simple: if a term matches both the exclusion list and the terminology, its ‘relevance’ field in the JSON output is set to ‘False’. This selected list of descriptors can be accessed and could help text miners to process other specialized corpora.

### Annotation process

#### Matchers descriptions

As described in the previous parts, the annotation process is based on a set of terminologies formatted in JSON and the articles to be annotated having undergone parsing processing. For each scientific article, the process analyses each sentence, token by token, to determine whether an annotation exists according to the terminologies used in SIBiLS.

The annotation process relies on four matchers:

- General Matcher: this matcher is used for all terminologies except those mentioned in the following points. It is based on a complex process: gene or disease annotations often exhibit syntax variations (‘BRAF’ gene written as ‘BRAF’, ‘B RAF’, or ‘B-RAF’). In gene and gene product terminologies, the syntax BRAF is listed, indicating that basic matching would not capture papers with the B-RAF annotation. To enhance recall, one step in the General Matcher consists to remove special characters and spaces both in the sentence content and terminology terms. Removing these characters aims to prevent potential matches from being missed due to variations in term formatting.
- Strict Matcher: focused on chemical formulas like SMILES or IUPAC names, this matcher applies a strict matching approach, including alphanumeric characters and symbols like dash, bracket, ….
- Accession Numbers Matcher: specific to the accession number terminology, the approach involves pattern matching where the regular expressions included into the terminology are compared to each token (or word) within sentences of documents.
- Grants Matcher: This matcher operates similarly to the strict matcher but with a nuanced distinction. It is triggered only when the sentence in the document includes lexical patterns indicative of grant citation, such as ‘funded by’. The only terminology used for the matching step is the ‘crossref funder registry’.

#### Structure of the annotations

**Figure 3:**
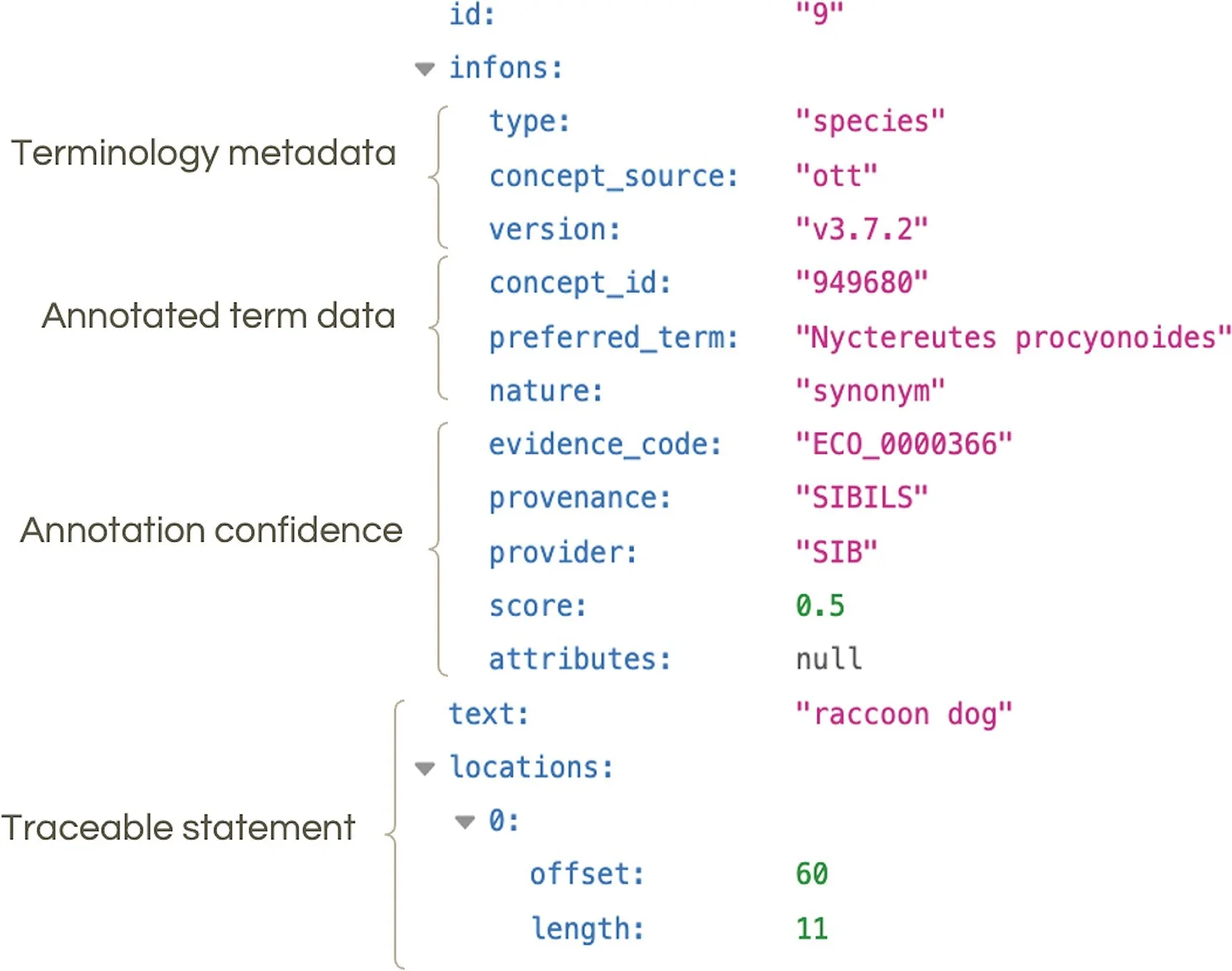
Structure of an annotation. Several fields are used to describe an annotation, related to the terminology used, the concept annotated, and the evidence details. The final output includes a MongoDB collection, an ElasticSearch index, and FTP archives containing the original documents, sentence-split versions, and generated annotations. These resources provide detailed information about each document and enable efficient data export.

The annotations are organized into several fields (Fig. 3) attached to BioC infons. Some fields pertain to the terminology used for creating the annotations, including the concept_source, which specifies the name of the terminology, its version, and an arbitrary type describing the kind of data (e.g. species for ott, gene for uniprot_swissprot…). Other fields relate to the matched concept: the concept_id, the preferred_term provided by the terminology, and nature, which indicates if the text matched (named « text », outside of the infons) in the document is a synonym of the preferred_term or the preferred_term itself.

Finally, the last fields provide information about the created evidence. When provenance is ‘SIBILS’, the ECO code, provider, and score are by default ‘ECO_0 000 366’ (‘evidence based on logical inference from automatic annotation used in automatic assertion’), ‘SIB’ and ‘0.5’, respectively. Those can be modified depending on the provenance, e.g. when the annotation is provided by third-party services derived from manual curation like ‘Plazi’ (another provenance and provider), the code will be replaced by ‘ECO_0 007 631’ (« computational inference used in manual assertion »), and score by ‘1.0’, which expressed the highest level of confidence.

### Evaluation

#### General annotations

We performed an analysis to assess the quality of the annotations based on a benchmark comprising 50 random MEDLINE abstracts. We extracted and evaluated all annotations found within these abstracts. Data are available on Zenodo (10.5281/zenodo.15112068).

The evaluation was based on two parameters:

- the first was depending on the quality of the annotation process,
- the second was based on the pertinence between the definition in the ontology and the context in the document.

Our output was a list of annotations linked to the corresponding pair paper-passage and a score (0 or 1), 0 for a false-positive annotation, 1 for a true-positive one.

For illustrative purposes, an example of a true-positive annotation and a false-positive annotation is presented: PMID29122328, sentence ‘Lobectomy for treatment of differentiated thyroid cancer: can patients avoid postoperative thyroid hormone supplementation and be compliant with the American Thyroid Association guidelines?’ The term ‘thyroid’ is assessed as a true positive as a MeSH term (D013961, definition: Thyroid Gland), but as a false positive annotation as a ChEBI term (CHEBI:9584, where ‘Thyroid’ is a synonym of ‘desiccated thyroid extract’) because in the context of the article, it refers to the thyroid gland rather than to pharmaceutical products.

We conducted an in-depth analysis to assess how the length of individual terms influences the quality of annotation, examining the potential effects and implications of varying term lengths on the quality.

## Results

### Overview of SIBiLS volumes

At the end of 2025, SIBiLS contained almost 80 million files across four collections, and ~16 billion annotations. On average, each document (or file in the case of supplementary data) contained around 397 annotations (Table 2).

**Table 2:** Volumes of SIBiLS contents (December 2024).

Collection	Nb documents	Nb annotations	Avg (anns/doc)
Medline	39 992 127	3 399 330 813	85
PMC	8 025 320	10 946 536 468	1364
Plazi	909 003	72 720 245	80
Supplementary data	29 656 484	1 779 389 038	60
	78 582 934	16 197 976 564	

This table summarizes the key statistics for each collection (Medline, PMC, Plazi and supplementary data) into SIBiLS. The column ‘Nb documents’ indicates the total number of documents in each collection; ‘Nb annotations’ represents the total number of annotations associated with each collection; ‘Avg (anns/doc)’ shows the average number of annotations per document, highlighting variations in annotation density across collections. Higher averages (e.g. PMC) indicate more detailed annotation per document, whereas lower averages suggest fewer annotations relative to document count. While the PMC full-text articles are unsurprisingly annotation richer, it is worth observing that the collection of supplementary data files is roughly equivalent to Medline.

### Evaluation of annotation accuracy

In the benchmark comprising 50 randomly selected abstracts from MEDLINE, a total of 5897 annotations were assessed (Table 3). Out of the total annotations, 5281 were categorized as positive, accounting for almost 90%, while 616 annotations were classified as negative, constituting 10% of the total. This leads to a precision of 0.90, and we assume that the relative recall is 1.0. Further, we assessed the inter-annotator agreement on a random subset of 100 annotations selected from the initial dataset. An inter-annotator agreement of 86% was measured, confirming the consistency and reliability of the produced annotations.

**Table 3:** Summary of annotations statistics.

Annotations (total number)	5.897
Avg of annotations/paper	118
Nb of true positive annotations	5.281
Avg of true positive anns/paper	106
Proportion of true positive anns (%)	89.55
Nb of false positive annotations	616
Avg of false positive anns/paper	12
Proportion of false positive anns (%)	10.45
Precision	0.9
Recall (relative)	1.0

The table provides an overview of the annotation performance, including a total of 5897 annotations with an average of 118 annotations per paper. Out of these, 5281 are true positive annotations, averaging 106 per paper and accounting for 89.55% of the total annotations. False positive annotations number 616, averaging 12 per paper and representing 10.45% of the total. The system achieves a precision of around 0.9.

Further, we observe a correlation among annotated term length and annotation precision. We observed that as the length of the terms increases, the percentage of true positives rises, while the proportion of false positives decreases (Table 4).

**Table 4:** Performance metrics across different term lengths.

	Terms length				
	*n* = 3	*n* = 4	*n* = 5	*n* = 6	*n* > 6
True positive	127	179	400	412	4163
False positive	106	65	72	98	275
Total	233	244	472	510	4438
True positive (%)	54.51	73.36	84.75	80.78	93.80

The table presents the number of true positives, false positives, and total annotations for various term lengths (*n* = 3, *n* = 4, *n* = 5, *n* = 6, *n* > 6), along with the corresponding percentages of true positives, highlighting improved accuracy with increasing term length.

**Figure 4:**
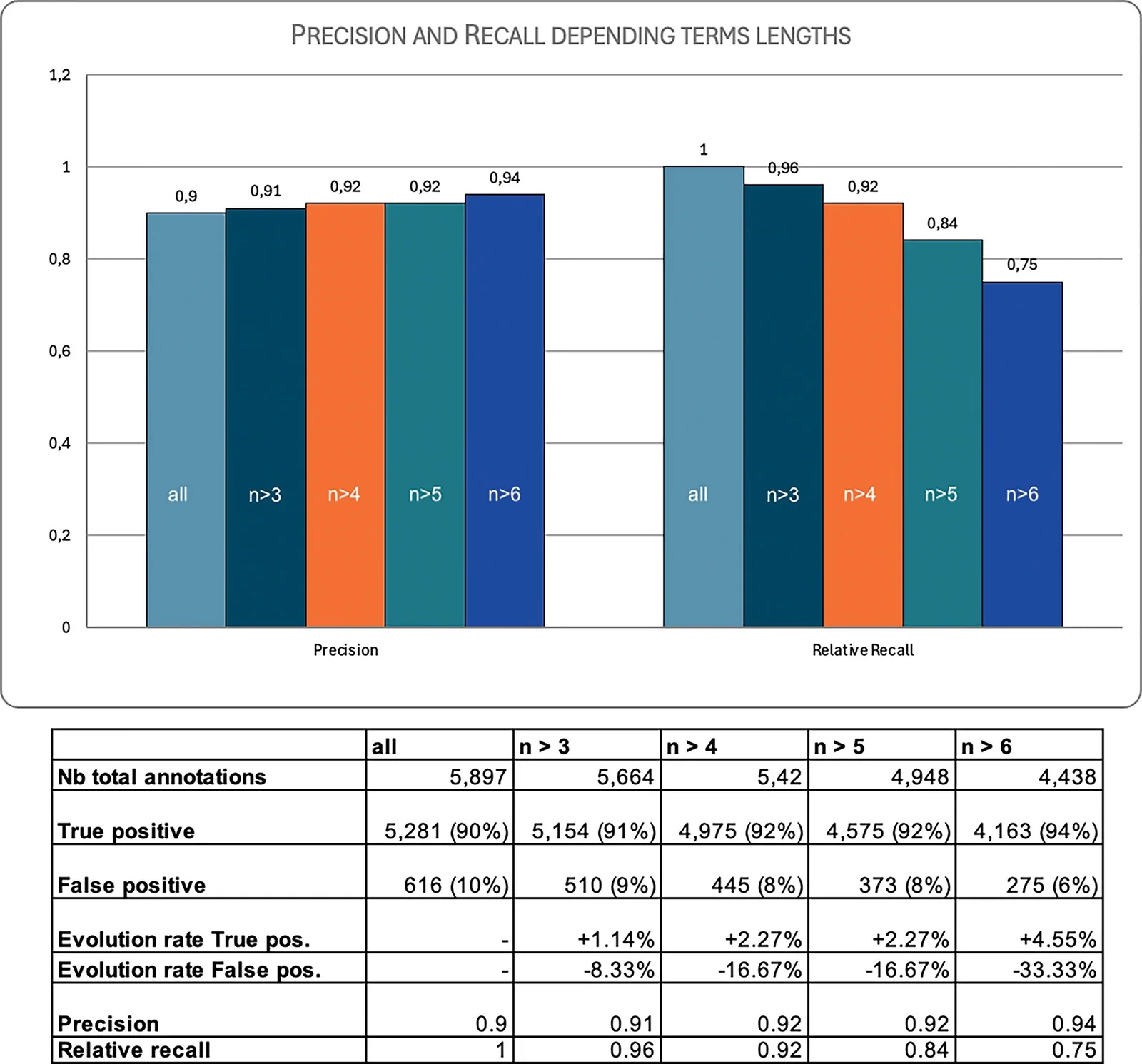
Precision and recall based on term lengths. Bar chart comparing precision and recall values across different minimum term lengths (*n* > 3, *n* > 4, *n* > 5, *n* > 6), illustrating how performance metrics evolve as term length increases.

This trend results in higher precision (0.94 instead of 0.9) but is concurrent with a decline in relative recall (0.75 instead of 1.0) (Fig. 4). Although precision is already high, this illustrates it could be relevant to determine a minimal length to avoid false positive annotations. The optimal length would be *n* > 6, with an evolution rate of +4.49% for true positives annotations, respectively.

However, for some terminologies, limiting the minimum length seems obviously problematic, notably for UniProtKB/SwissProt dedicated to gene and protein names; therefore, in addition to the term length, it is interesting to consider the source terminology upon which the annotation is built.

**Figure 5:**
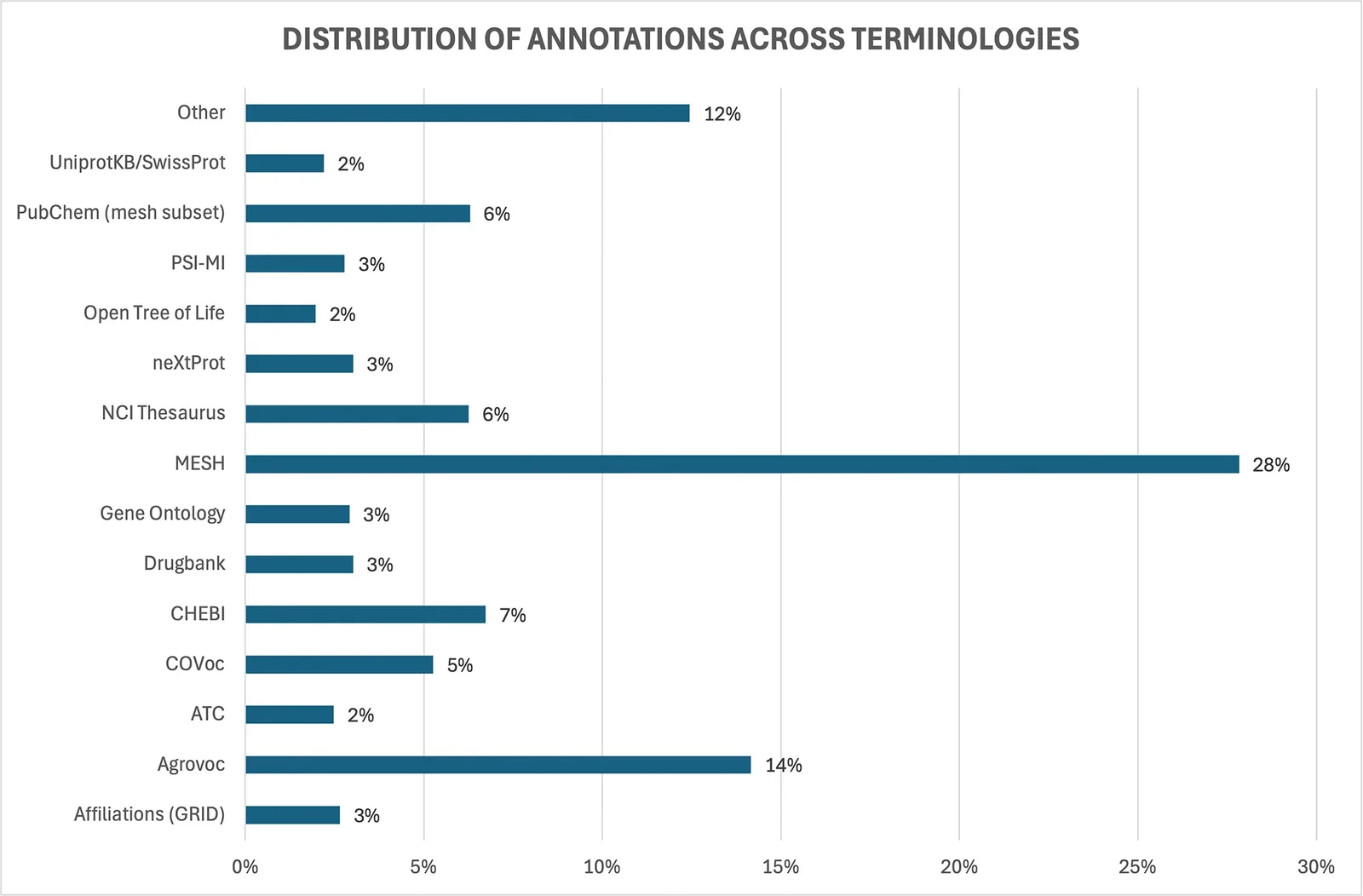
Distribution of annotations across terminologies. A histogram illustrating the proportion of annotations assigned to each terminology and corresponding percentages. ‘Other’ is about terminologies with <2% of annotations.

In Fig. 5, we provide an overview of how annotations are distributed across the various terminologies included in the dataset. By examining this distribution, it would be easier to better understand, which terminologies are most heavily represented and identify potential areas for further refinement.

Having established the distribution of annotations across the terminologies, highlighting the dominant ones, we next focused on analysing the proportion of true and false positive annotations for each terminology. Table 5 highlights on which terminologies we could focus our improvement effort.

**Table 5:** Percentage of true and false positives associated with each terminology.

Terminology	Nb annotations	True (%)	False (%)
Affiliations (GRID)	156	100	0
Agrovoc	835	92.10	7.90
ATC	146	92.47	7.53
Cell Ontology	13	100	0
**Cellosaurus**	56	26.79	**73.21**
CHEBI	397	94.46	5.54
COVoc	310	94.19	5.81
Detection Methods	34	100	0
Disprot	105	100	0
Drugbank	178	95.51	4.49
ECO	10	100	0
**ENVO**	89	38.20	**61.80**
**FLOPO**	2	0	**100**
Gene Ontology (GO)	172	94.19	5.81
ICDO3	8	100	0
**License**	2	0	**100**
LOTUS	105	89.52	10.48
MDD	7	100	0
MeSH	1641	98.29	1.71
NCBI Taxonomy (Full)	102	93.14	6.86
NCBI Taxonomy (models)	24	95.83	4.17
NCI Thesaurus	369	76.96	23.04
neXtProt	178	83.15	16.85
Open Tree of Life (OTT)	116	93.10	6.90
**Plant Ontology (PO)**	43	44.19	**55.81**
PPI-PTM	27	85.19	14.81
PSI-MI	164	98.78	1.22
PubChem (subset MeSH)	371	78.44	21.56
**Extended RO (Relation Ontology)**	107	27.10	**72.90**
UniprotKB/SwissProt	130	82.31	17.69

Among the 30 terminologies assessed in this benchmark, six (in bold) exhibit a false positive rate exceeding 50%. This number can be reduced to four by removing ‘License’ and ‘FLOPO’ as their evaluation, based on only two terms, appears less significant for this terminology.

Six terminologies exhibit a false positive rate exceeding 50%. However, the limited number of terms evaluated for ‘FLOPO’ [12] and ‘License’ [41] (two terms) in the sample makes it difficult to draw any conclusive interpretation. Additionally, the Relation Ontology terminology, expanded by the team to meet the needs of the BiotXplorer service to reveal biotic interactions, will need to be revised to remove terms that introduce significant noise.

**Figure 6:**
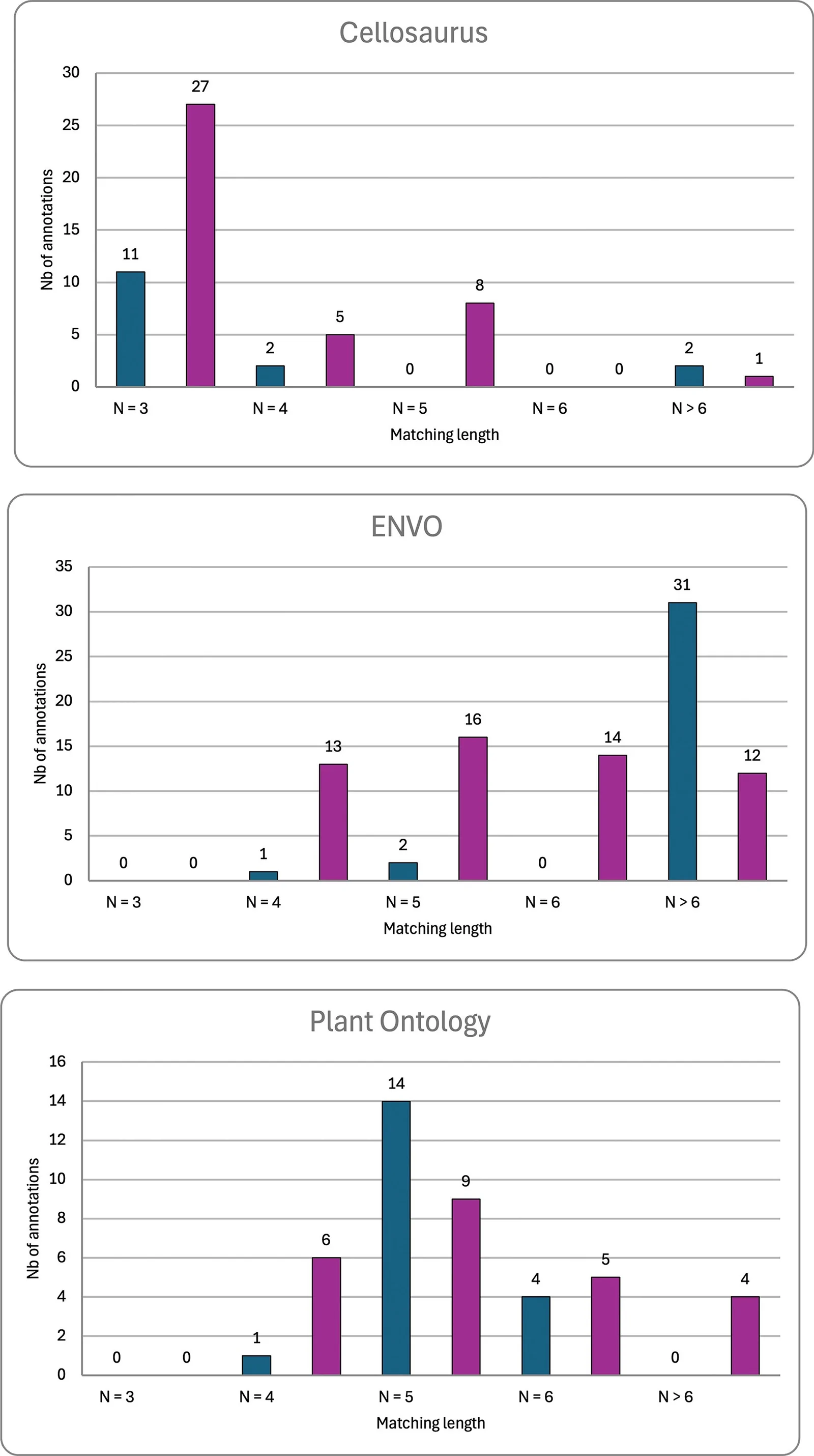
Distribution of true positive and false positive annotations by matching length for cellosaurus, ENVO, and Plant Ontology. This figure illustrates the distribution of true positive (blue bars) and false positive (purple bars) annotations across different matching lengths (*N*) for three terminologies: Cellosaurus, ENVO, and Plant Ontology. The *x*-axis represents the matching length categories, while the *y*-axis indicates the number of annotations. For Cellosaurus, annotations with shorter lengths (*N* ≤ 4) dominate but result in a high number of false positives. In ENVO, annotations with matching lengths greater than 6 (*N* > 6) show the highest precision. For Plant Ontology, a matching length of *N* = 5 provides a balance between true positives and false positives. These results emphasize the influence of the length parameter and suggest the need for additional semantic filtering to reduce false positives.

We conducted a detailed analysis of the distribution of true and false positive annotations based on their length on the three remaining terminologies (Fig. 6). This analysis provides insights into the optimal minimum length to apply, aiming to improve precision while maintaining sufficient recall.

For Cellosaurus [23], introducing a minimum length to filter out false positives might seem like a good idea, but it would result in missing most of the terms (11 out of the 15 true positives). Instead, manual curation of the CelloSaurus terminology by cleaning high-frequency terms is likely the most promising strategy to consider.

For ENVO [11, 12], implementing a minimum length, greater than six, initially appears to be an effective approach. However, further analysis reveals that the false positive terms are primarily associated with contexts unrelated to the environment, indicating that the issue lies in semantics rather than term length. To reduce the number of false positives, the most effective strategy could be to focus exclusively on articles related to environmental and biodiversity topics.

For Plant Ontology [14], applying a minimum length filter could be a viable approach, but setting it to *N* > 6 would eliminate all true positive annotations. A minimum length of *N* = 5 appears to be a more reasonable choice. Similar to ENVO, many false positive annotations are due to semantic issues rather than term length. Here again, focusing exclusively on articles related to the environment or biodiversity could significantly reduce the number of false positives, thereby improving overall precision.

## Discussion

Through this evaluation, we have identified several approaches to improve the accuracy of SIBiLS annotations, listed below:

- Specific matchers and parameters

This approach of developing specialized matchers aims to decrease the occurrence of false positives while increasing the number of true positive annotations. These matchers enable the application of matching parameters tailored to the specific terminology being annotated. After the successful implementations of specific matchers for chemical entities, identifiers, and grants, we envision adding a dedicated matcher for gene annotations.

- Accession numbers strategy

Crossed with an evaluation provided by the team, it would be relevant to change the strategy to match the accession numbers. Instead of filtering manually the ‘too permissive’ patterns, all regex patterns will be used, and database prefixes added (e.g. UNIPROT: *regex* or PDB: *regex*).

- Journal-specific terminology

To enhance the precision and uniformity of annotations, an additional approach involves establishing sorting criteria for annotated journals. Each journal, upon integration into SIBiLS, could be tagged based on its respective field (e.g. ecology, biodiversity, medicine, etc.). Alternatively, a classifier could be developed to categorize journals into subgroups.

Subsequently, depending on these tags or subgroups, all or only specific terminologies would be used in the annotation process. It makes little sense to create annotations from DrugBank terminology in a paper on ecology, or to annotate LOTUS terms (natural chemical entities) in astrophysics articles. However, certain broader terminologies or vocabularies, such as crossref funder registry (grants), SPDX (licenses), or accession numbers, would need to be universally applied across the entire article collection.

- Manual curation work

Manual curation work can also be incorporated to eliminate any lingering false positives in terminologies, even those that persist despite employing more refined matching parameters.

- High-frequency terms analysis

To supplement the manual curation process, another strategy could involve automatically generating a list of high-frequency terms for each terminology and applying a cut-off threshold. Any term reaching or exceeding this high-frequency threshold would then be appended to the exclusion list associated with the respective terminology.

- Named entity recognition

Another possibility would involve implementing NER, a component of NLP that categorizes named entities into predefined classes. This would help eliminate potential ambiguities in cases where two terms from different terminologies are identical, and the context permits only one annotation to be preserved.

## Conclusion

In this study, we examined how effectively terminological terms can be recognized in scientific papers. With a relative recall of 75%, the precision of the concept recognition module reaches 94%. These results are, however, very contrasted depending on the terminology. Further, our analysis reveals a direct relationship between recall, precision, and the length of the matched entities. Notably, we found that as the length of the matched term expands, recall diminishes while precision improves. Symmetrically, a reduction in term length tends to elevate recall but lower precision. This observation underscores the trade-off inherent in text-mining endeavours, emphasizing the need to meticulously weigh term length in both algorithmic development and assessment. While prioritizing precision is vital for guaranteeing the relevance and correctness of matched terms, it must be counterbalanced by preserving sufficient recall to encompass all pertinent terms within texts. Striking this equilibrium demands thoughtful evaluation of multiple factors like the characteristics of the terminologies. In the current settings of our annotation pipeline, all annotations are available via our APIs and can be visualized in the Graphic User Interface. We are considering the possibility to display only the annotations above a certain threshold, but further evaluation is needed as the threshold is dependent on various parameters.

In upcoming updates, our goal is to further improve SIBiLS, the annotation pipeline and the related services, such as Variomes and BiotXplorer, to better support the scientific community, particularly in fields like biomedicine and ecological sciences from biodiversity studies to taxonomy. Enhancing a scientific annotation (text-mining) service increases both the accuracy and the efficiency of extracting relevant information from the rapidly growing body of publications. Better annotations enable researchers to identify key concepts, relationships, and evidence more quickly, reducing the time spent on manual screening and improving literature reviews, data curation, and knowledge discovery. Such improvements make scientific information more accessible, structured, and reusable, which benefits both researchers and the broader scientific community. For instance, through a collaboration with publishers, we plan to integrate new collections, including open-access biodiversity journals that are currently unavailable in PMC. In addition, optimization about matching services, including a minimal length depending on which terminology is used, will be tested and should result in a better global quality of SIBiLS annotations and related services.

## Notes

SIBiLS: https://sibils.org/

BiotXplorer: https://biotxplorer.sibils.org/

Variomes: https://variomes.sibils.org/
